# Reply to Otiti-Sengeri et al. Chorioretinitis among Immigrant and Travellers. Comment on “Mansour et al. Presumed Onchocerciasis Chorioretinitis Spilling over into North America, Europe and Middle East. *Diagnostics* 2023, *13*, 3626”

**DOI:** 10.3390/diagnostics14050479

**Published:** 2024-02-23

**Authors:** Ahmad Mansour, Linnet Rodriguez, Hana Mansour, Madeleine Yehia, Maurizio Battaglia Parodi

**Affiliations:** 1 Retina Service, Department of Ophthalmology, American University of Beirut, Beirut 1107, Lebanon; 2 Retina Service, Wills Eye Hospital, Thomas Jefferson Medical Center, Philadelphia, PA 19107, USA; linnetr200@gmail.com (L.R.); hanamansour100@gmail.com (H.M.); 3 Retina Service, University of Illinois Chicago, Chicago, IL 60612, USA; madeleineyehia@gmail.com; 4 Retina Service, Department of Ophthalmology, Ospedale San Raffaele, University Vita-Salute, 20132 Milan, Italy; maubp@yahoo.it

The comments by Otiti et al. [1] are well taken. These comments were raised by us as authors. To come up with a presumed diagnosis of ocular onchocerciasis without anterior segment disease, without cutaneous lesions, and without serology is indeed intriguing and requires a kind of artificial intelligence to rule out other similar diseases. All four patients in the study resided in Africa for decades, frequented the riverside at least weekly, and gave a history of blackfly bites, but none suffered skin lesions. The clinical progression of the disease over decades, such as subretinal tracts, peripapillary involvement, etc., documented by multimodal imaging was the key factor in our presumed diagnosis. We compared these findings with almost every tropical disease, and we could only match the symptoms with ocular onchocerciasis. We extensively searched old atlases and old papers on tropical diseases to see fundus changes. The search was not easy, as most papers were related to epidemiology (field work) or infectious diseases, with a few ophthalmic papers showing fundus photographs. Basically, we agree that what we presented is either isolated ocular onchocerciasis or a new entity mimicking ocular onchocerciasis; hence, we have chosen the term “presumed” ocular onchocerciasis [2]. Tuberculosis, sarcoidosis, toxoplasmosis, syphilis, and other diseases can manifest uniquely in the eye without other bodily signs or symptoms [3]. Our aim was to increase awareness of the ophthalmic findings in tropical diseases. There is a big lacuna in the in the literature and in retina atlases concerning fundus pictures. Our colleagues from Ghana and South Sudan can fill this gap by submitting typical onchocerciasis chorioretinitis photographs to the major retina websites.
